# Revisiting Stress “Deafness” in European Portuguese – A Behavioral and ERP Study

**DOI:** 10.3389/fpsyg.2018.02486

**Published:** 2018-12-10

**Authors:** Shuang Lu, Marina Vigário, Susana Correia, Rita Jerónimo, Sónia Frota

**Affiliations:** ^1^School of Foreign Languages, Renmin University of China, Beijing, China; ^2^Centre of Linguistics, School of Arts and Humanities, University of Lisbon, Lisbon, Portugal; ^3^NOVA CLUNL, Faculty of Social Sciences and Humanities, Universidade NOVA de Lisboa, Lisbon, Portugal; ^4^Centro de Investigação e de Intervenoção Social (CIS-IUL), Instituto Universitário de Lisboa (ISCTE-IUL), Lisbon, Portugal

**Keywords:** European Portuguese, stress deafness, vowel reduction, ERPs, mismatch negativity, working memory

## Abstract

European Portuguese (EP) is a language with variable stress, and the main cues for stress are duration and vowel reduction. A previous behavioral study has reported a stress “deafness” effect in EP when vowel quality cues are unavailable. The present study recorded both event-related potentials (ERPs) and behavioral data to examine the stress processing by native EP speakers in the absence of the vowel quality cues. Our behavioral result was consistent with previous research, showing that when vowel reduction is absent EP speakers demonstrated a stress “deafness” effect similar to that found in speakers of languages with fixed stress or without any lexical stress marking. In the ERP task, both the trochaic and iambic conditions yielded mismatch negativity (MMN) and late negativity, suggesting that EP speakers are able to discriminate the two stress patterns without vowel reduction at the pre-attentive stage. Moreover, the ERP and behavioral data revealed compatible results regarding the pattern of stress bias in EP. In the EPR task, the MMN and late negativity components were more negative and span over a larger temporal window in the iambic condition than in the trochaic condition, indicating a higher sensitivity for the iambic stress pattern. In the behavioral task, EP speakers responded more accurately and more quickly to the iambic stress. These results match recent developmental findings in the acquisition of stress, but speak against the dominant view in EP phonological literature which assumes penultimate stress to be the regular stress pattern. In addition, both the ERP and the behavioral data showed that EP speakers’ stress processing was influenced by their working memory (WM) capacity. The participants with high WM capacity outperformed the participants with limited WM capacity in the iambic condition. In sum, our results broaden the current knowledge on stress processing by EP speakers at both the pre-attentive and attentive levels.

## Introduction

Lexical stress refers to the prominent syllable in a word. Some languages have fixed stress, meaning that stress always falls on a particular position. For example, Finnish and Hungarian words are always stressed on the first syllable (Tompa, 1972; Karlsson, 1999), and Polish words mostly on the penultimate syllable (Comrie, 1967). Other languages (e.g., English, Spanish, and German) have variable stress, meaning that the position of stress in a word is not predictable. In these languages word stress can convey lexical distinctions and there are minimal pairs that only differ in stress pattern (e.g., *insight*/’InsaIt/ vs. *incite/*In’saIt/ in English). Thus, the processing of word stress is particularly relevant in the use of such languages. Previous studies have shown that compared with speakers of languages with variable stress, speakers of languages with fixed stress have difficulties in distinguishing non-words that differ only in stress pattern (e.g., Dupoux et al., 1997, 2001, 2008; Peperkamp et al., 2010; Domahs et al., 2012), and this result was not influenced by the degree of transparency between stress locations and word boundaries (Rahmani et al., 2015, but see Peperkamp, 2004). Moreover, lexical stress is typically signaled by acoustic cues such as duration, pitch (fundamental frequency, F0), intensity and vowel quality (Fry, 1958; Bolinger, 1961; Lehiste, 1970). Languages differ in the weighing of these phonetic cues and the absence of certain cues may influence listeners’ perception of stress (e.g., Sluijter et al., 1997; Cooper et al., 2002). For instance, in English the primary cue for stress is relative pitch prominence (e.g., Fry, 1958; Morton and Jassem, 1965), which outranks intensity, duration and vowel quality (Beckman, 1986; Sluijter and van Heuven, 1996). However, in Catalan, syllable duration, spectral tilt and vowel quality have been found to be the reliable acoustic correlates of stress differences (Astruc and Prieto, 2006; Ortega-Llebaria and Prieto, 2010).

European Portuguese (EP) is a language with variable stress, and the stress may fall on any of the last three syllables. Pitch has been considered as a low correlate of stress in EP, due to the sparse distribution of pitch accents (Vigário and Frota, 2003). Specifically, in EP not every stressed syllable gets a pitch accent, and words are pitch accented mostly in nuclear position (Frota, 2002, 2014). Duration was reported as a crucial cue for word stress, particularly in the absence of vowel reduction (Delgado-Martins, 1977, 1986; Andrade and Viana, 1989). Vowel quality cues, however, have been claimed to be the primary cue for the perception of stress in EP. Castelo (2005) reported that EP speakers usually identified full vowels as stressed, even though they were unstressed. Correia et al. (2015) found that although duration is a prosodic cue of word stress in EP, it is not sufficient for EP speakers to process stress contrasts in the absence of vowel reduction. Correia et al. (2015) conducted a series of discrimination and sequence recall tasks with stress and phoneme contrasts to investigate EP speakers’ perception of word stress. The results showed that without the vowel quality cues, EP speakers exhibited a stress “deafness” effect similar to that found in speakers of languages with fixed stress, while demonstrated near-perfect performance to the phoneme contrasts. In the presence of vowel reduction, the stress contrasts elicited similar error rates as the phoneme contrasts and no stress “deafness” was found. Rahmani et al. (2015) extended the research on stress “deafness” to languages without lexical prosodic markings, and claimed that stress “deafness” is a property of speakers of languages without lexical stress or accent markings. However, Correia et al.’s (2015) results seem to suggest that vowel reduction may take over the prosodic lexical markings in the attentive processing of stress by EP speakers.^1^

In order to thoroughly investigate whether speakers of EP can pre-attentively and attentively discriminate CVCV pseudo-words that only differ in stress pattern (i.e., trochee vs. iamb) in the absence of vowel reduction, the present study recorded participants’ event-related potentials (ERPs) and behavioral responses. Previous research has suggested that perceptual discrimination may occur at the pre-attentive stage, but not (yet) at the attentive/behavioral level (e.g., Kraus et al., 1995; Tremblay et al., 1998). Moreover, previous studies on Turkish demonstrated divergent results between behavioral and ERP methods. Turkish has a system of fixed stress on the final syllable (e.g., Sezer, 1983; Lewis, 2000; Kabak and Vogel, 2001). Altmann (2006) found that Turkish participants had problems identifying stress positions in a behavioral stress perception task, whereas Domahs et al. (2013) reported that Turkish speakers were not “deaf” for lexicalized stress patterns in an ERP study. So we might expect that native speakers of EP may be able to discriminate the two stress patterns without vowel reduction at the pre-attentive stage in ERPs, even though they demonstrated a stress “deafness” effect in behavioral tasks as shown in Correia et al. (2015).

To our knowledge, no study has been conducted to examine the pre-attentive processing of stress by native speakers of EP. Previous electrophysiological studies have been performed on other languages with either fixed or variable stress, such as Polish, Turkish, Cairene Arabic and German (Knaus et al., 2007; Domahs et al., 2008, 2012, 2013; Domahs U. et al., 2014). These studies utilized a stress violation paradigm, in which participants were presented with correctly and incorrectly stressed words and were required to judge whether the stress was assigned to the appropriate syllable. The results showed that even speakers of languages with fixed stress were sensitive to most violations, and they displayed different neural reactions to the violations involving default and exceptional stress patterns. To investigate the stress processing by German adults and infants, Weber et al. (2004) used a passive oddball paradigm which did not require participants’ attention while their brains’ responses to the stress patterns were recorded. The results showed that the German adults exhibited mismatch negativity (MMN) to both the trochaic and iambic stress patterns, while the 5-month-old infants only displayed a significant mismatch response (MMR) to the trochaic stress pattern, which is more frequent (and arguably the default pattern) in the language. In the present study, we also employed the passive oddball paradigm to examine the pre-attentive processing of stress by EP speakers. In addition, the participants received a separate behavioral task, which provided data regarding their attentive responses after they fully processed the stress patterns. Therefore, a passive ERPs task combined with a separate behavioral task can give us a thorough view of how native speakers of EP attentively and pre-attentively process stress without vowel quality cues. We focused on two ERP components which have been claimed to be relevant to automatic auditory perception. The MMN is a negative wave elicited by the deviant stimuli in a sequence of frequently presented stimuli. The MMN usually peaks at about 100–300 ms after the onset of deviant stimuli (may vary slightly according to different paradigms and type of deviant stimuli) and has a prominent frontal distribution (Näätänen et al., 1993, 2004). The late negativity is another negative wave that occurs around 350–600 ms after the onset of deviant stimuli. This component has been associated with neural processes of auditory rule extraction (e.g., Zachau et al., 2005). If native speakers of EP are able to discriminate stress in the absence of vowel reduction, they would show MMN and late negativity to both the trochaic and iambic conditions.

Another question that was investigated in the present study is whether the EP speakers would show asymmetrical performance for trochaic and iambic stress patterns. Chrabaszcz et al. (2014), in a behavioral study, found that pitch and vowel quality cues were more influential for stress identification in the iambic pattern than in the trochaic pattern. The authors attributed this result to an expectation bias toward the trochaic stress in the languages tested in the experiment (i.e., the iambic stress would be most different from the expectation). Some electrophysiological studies have also shown that the frequency of stress patterns influences the neural processing of word stress by native speakers of languages with fixed stress. For example, Polish is a language with fixed penultimate stress and some well-defined exceptions. Using a stress violation paradigm, Domahs et al. (2012) found that native speakers of Polish showed a task-related P300 effect to the violations involving the exceptional stress patterns, but not to the violations involving the default penultimate stress pattern, despite the fact that they exhibited difficulties to reject any kind of stress violation in the behavioral task. Using the same paradigm, Domahs et al. (2013) reported similar results on Turkish, which has final stress by default and some exceptional non-final stresses. Turkish speakers exhibited an N400-like effect to incorrect final stress, while a P300 effect to incorrect penultimate and antepenultimate stress. These results were interpreted to indicate that speakers of languages with fixed stress might be less sensitive to the default stress pattern than to the exceptional patterns. If EP speakers behave similarly to the speakers of languages with fixed stress, in the absence of the vowel quality cue, we may expect them to show processing difficulties to both the trochaic and iambic stress patterns in the behavioral task and to be less sensitive to the more frequent stress pattern in the EPR experiment.

According to the database of Frequency Patterns of Phonological Objects in Portuguese (FrePOP, Frota et al., 2010), 74% of Portuguese words with two or more syllables have penultimate stress, 23% of words have their stress on the final syllable and 3% of words are stressed on the antepenultimate syllable. Thus, the dominant view in the phonological literature on EP assumes penultimate stress to be the regular stress pattern (Viana et al., 1996). However, if we consider the token frequency and take monosyllabic words into account^2^, the distributions of penultimate stress and final stress become very close (with final stress above 40%), and the final stress is even more frequent than the penultimate stress if the computation is based on disyllabic words only (Frota et al., 2006; Vigário et al., 2010). Thus, no final conclusion has yet been reached on whether penultimate stress or final stress is the predominant stress pattern in EP. EP is not the only language that has contradictory accounts regarding the default stress pattern. For instance, Russian is also a language with variable stress. Unlike many previously discussed languages that have a clearly dominant stress pattern, there is no more frequently occurring stress pattern in Russian disyllabic words (Jouravlev and Lupker, 2014). Even though the dominant view in the phonological literature on Russian assumes iamb to be the default stress pattern, behavioral and electrophysiological studies have shown conflicting results. Molczanow et al. (2013) employed ERP measures and revealed that trochaic stress is less costly than iambic stress in the prosodic processing by native Russian speakers. Nevertheless, using behavioral measures Crosswhite et al. (2003) found support for an iamb default. If EP speakers behave similarly to Russian speakers, we may find divergent results in the behavioral and ERP tasks with respect to asymmetrical processing of stress patterns.

Alternatively, EP speakers may display consistent results between the behavioral and ERP methods pertaining to the stress processing patterns, as suggested by several developmental studies by infants whose native languages have variable stress. As mentioned previously, English, German, and Dutch, unlike French, are languages with variable stress. In these languages trochaic stress is more frequent than iambic stress (e.g., Cutler and Carter, 1987; Jessen, 1999; Sebastián-Gallés et al., 2000). Using a Head-turn Preference Procedure (HPP), previous research has shown that infants whose native language is English, German or Dutch preferred to listen to (non-)words with a trochaic stress pattern over (non-)words with an iambic stress pattern (e.g., Jusczyk et al., 1993; Echols et al., 1997; Höhle et al., 2009; Dekker, 2015). By contrast, infants whose native language is French, which has fixed stress and the stress always falls on the last syllable of words or phrases, showed no preference for either of the stress patterns. In an ERP study using an oddball paradigm, Friederici et al. (2007) reported that 4-month-old German infants revealed a positive mismatch response (MMR) when the deviant stimulus was iambic, but not when it was trochaic. To the contrary, 4-month-old French-learning infants demonstrated a mismatch response when the deviant stimulus was a trochaic disyllable, but not when it was an iambic one. The positive MMR has been interpreted as a less mature discrimination response specific to infants, and it has been claimed to reflect an acoustic form of analysis rather than a more abstract processing (Morr et al., 2002; Trainor et al., 2003; Rivera-Gaxiola et al., 2005). Because the positive MMR has also been associated with additional effort in the perceptual processing of deviant stimuli, the enhanced effect in the 4-month-old German infants indicates that the memory structures for the iambic stress pattern are less well established than those for the trochaic stress pattern, and vice versa for the French infants (Friederici et al., 2007). Therefore, the Friederici et al. (2007) results are indeed compatible with the above-mentioned behavioral studies, showing that German infants acquired trochaic stress earlier than iambic stress. Using the same oddball paradigm, Weber et al. (2004) found that 5-month-old German infants displayed a MMN to the trochaic deviant, but not to the iambic deviant stimulus. This result suggests that by 5 months German infants no longer exhibit enhanced processing effort for the less frequent stress pattern, but instead show a more mature response to the typical stress pattern of their native language. Besides, Weber et al. (2004) also noticed that the infant results are different from the findings for adults, which revealed MMN for both trochaic and iambic stress patterns. In short, the developmental behavioral and ERP studies on infants whose native language has variable stress suggest a processing advantage for the stress pattern typical of the native language.

Taken together, previous studies seem to indicate that native speakers of languages with fixed stress may not differentiate stress patterns at the behavioral level, but may be more sensitive to the uncommon stress pattern (which needs more effort to process) at the electrophysiological level. However, native speakers of languages with variable stress tend to prefer the predominant or regular stress pattern at the behavioral level and may expose a processing advantage (possibly shown by an asymmetrical effect) for the same pattern at the electrophysiological level. The complexity of the problem suggests that in order to better understand the relationship between effects found in the behavioral and electrophysiological data, parallel studies at both levels are clearly needed. Thus, our study reassesses this issue in adult native speakers of EP using both behavioral and electrophysiological methods. If in the absence of vowel quality cues EP speakers behave similarly to the native speakers of languages with fixed stress, we might find no advantage of either the trochaic or iambic stress pattern behaviorally, but an increased sensitivity to the less frequent stress pattern in the electrophysiological data, due to the enhanced processing effort. Nevertheless, if EP speakers behave similarly to the speakers of languages with variable stress, we would expect them to show an advantage for the more frequent stress pattern in the behavioral task (contrary to previous findings in Correia et al., 2015), and to display either an MMN for both stress patterns (as the German adult speakers), or an asymmetrical effect pointing to one of the stress patterns, which should be easier to process.

Besides, previous research has shown that stress processing depends crucially upon memory load (e.g., Dupoux et al., 1997, 2001; Peperkamp et al., 2010; Domahs F. et al., 2014; Heisterueber et al., 2014). For example, Dupoux et al. (1997) found that in an ABX task French participants had significantly more difficulties in lexical stress discrimination than Spanish participants, while in an AX task French participants could accurately detect the acoustic correlates of lexical stress. Dupoux et al. (1997, 2001) claimed that the French participants’ difficulties in the ABX task should not be attributed to their perceptual capacities, instead these difficulties should lie in short-term memory. Stress distinctions play no lexical role in French, and are hence more difficult to be recorded in a short term memory store for the French participants than for the Spanish participants, particularly in more demanding tasks. These results demonstrated the role of short-term memory in stress abilities. Heisterueber et al. (2014) also reported that at both the behavioral and neuro-functional levels German speakers exhibited inter-individual differences in word stress processing, which might be attributed to individual working memory (WM) span. Moreover, in a non-word reading task Domahs F. et al. (2014) found that German speakers’ individual WM capacity was positively correlated with their assignment of main stress to the antepenultimate syllable, and negatively correlated with the assignment of stress to the final syllable. However, there was no significant correlation between WM capacity and stress assignment to the penultimate syllable, which has been claimed to be the default stress pattern in German (e.g., Wiese, 1996). Domahs F. et al. (2014) argued that this pattern of results supported the assumption of leftward stress processing in German, according to which, the antepenultimate stress is more cognitively demanding than the ultimate stress for German speakers. Therefore, participants with good WM capacity were able to use antepenultimate stress, while participants with limited WM capacity tended to avoid it and use more ultimate stress. In the present study, we assessed EP speakers’ WM capacity through a forward and backward digit span task, and then divided the participants into two groups according to their WM performance, in order to further investigate how WM span would influence the stress processing of EP speakers at the behavioral and neurophysiological levels.

In sum, the present study collected both behavioral and electrophysiological data to investigate stress processing by EP speakers. Three main research questions were addressed: (1) Can EP speakers pre-attentively and attentively discriminate CVCV pseudo-words with trochaic and iambic stress patterns in the absence of vowel reduction? (2) Will EP speakers show asymmetrical performance for the trochaic and iambic stress patterns? (3) How does WM ability influence stress processing by EP speakers at the behavioral and electrophysiological levels?

## Experiment 1

Experiment 1 included a WM task and an ERP task. EP speakers were divided into two groups based on their performance in the WM task. The aim of the current experiment was to examine EP speakers’ pre-attentive discrimination of trochaic and iambic stress patterns in the absence of vowel quality cues, and whether the pre-attentive stress processing was influenced by different WM capacity.

### Methods

#### Participants

Twenty native speakers of EP (6 males and 14 females) were recruited in the present study. All participants were students at the University of Lisbon, and were between the ages of 18 and 32 years old (*M* = 21.95, *SD* = 4.10). They were right-handed according to the Edinburgh Handedness inventory (Oldfield, 1971), and reported having normal vision and hearing. None of them had history of speech or neurological impairment. Another four participants were recruited, but were excluded from data analysis due to technical problems or not being able to complete the entire experiment. All participants received either course credit or a voucher for their participation.

#### Materials

**FIGURE 1:**
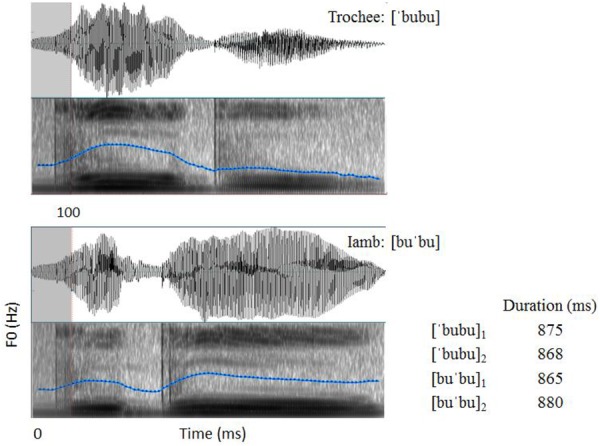
Spectrograms of the trochaic and iambic stress patterns. Physical differences start at 100 ms.

The stimuli in Experiment 1 were the same as the stimuli used in Lu et al. (2016). The disyllables [bubu] with either a trochaic or an iambic stress pattern were naturally produced by a female native speaker of EP. Each of the stress patterns was produced twice, resulting in four stimuli in total ([’bubu]_1_, [’bubu]_2_, [bu’bu]_1_, and [bu’bu]_2_). The stimuli were pseudo-words in EP and were recorded at a sampling rate of 22050 Hz. The mean durations of the trochaic and iambic tokens are 872 ms and 873 ms, respectively. Following Weber et al. (2004), the first 100 ms of [’bubu]_1_, [’bubu]_2_, and [bu’bu]_2_ were replaced by the first 100 ms of [bu’bu]_1_, in order to control for the acoustic onset differences. After the manipulation, no pitch discontinuity was observed in any of the stimuli (see Figure 1). Three native speakers of EP who did not participate in the experiment judged all the stimuli as perceptually natural.

#### Procedure

All participants were firstly tested on auditory WM through a forward and backward digit span task, in which they heard digit sequences and were asked to repeat the numbers in forward and reverse order. The forward span begins with a length of three digits and the backward span starts with a length of two digits. The digits were presented by an experimenter at a rate of one per second and two trials were presented at each increasing sequence length. Testing stopped when the participant failed to accurately repeat both trials at one sequence length or when the maximal sequence length was reached (nine digits for forward span and eight digits for backward span).

Then the participants did the ERP experiment, in which they were watching a muted movie (*The Gold Rush* by Charlie Chaplin) in a sound-attenuating booth while the stimuli were presented through a loudspeaker at a constant and comfortable hearing level. The participants were asked to ignore the sounds and focus on the movie, and they were given comprehension questions regarding the movie after each block. All participants answered at least 75% of the questions correctly. Two types of blocks were created in a passive oddball paradigm: (1) Trochaic block: the iambic tokens were presented as standards, while the trochaic tokens served as deviants; and (2) Iambic block: the frequently occurring trochaic tokens were occasionally replaced by the deviant iambic tokens. Within each block each token of the deviants and standards were presented 50 times and 250 times, respectively, resulting in 600 trials in total (50 × 2 tokens plus 250 × 2 tokens). The stimuli were presented in a pseudo-random order, with at least two standards preceding each deviant. We selected 100 clean standards (50 × 2 tokens) that were not immediately preceded or followed by any deviants in each block to compare with the same acoustic stimuli used as deviants in the other block. The offset-to-onset inter-stimulus interval randomly varied between 800 and 850 ms to prevent participants’ automatic anticipation of stimulus onset. In order to avoid participant fatigue, each block was split equally into two sub-blocks, with each one lasting for about 8 min. The order of the four sub-blocks was counterbalanced across participants. Before the experimental blocks, the participants received a practice block, in which each token of the two stress patterns was equally presented for 75 times. The practice block was excluded from data analysis. Stimulus presentation was controlled by E-Prime 2.0 software (Psychology Software Tools, Pittsburgh, PA, United States).

Continuous EEG was recorded from 29 Ag/AgCl scalp electrodes according to the international 10-20 system of electrode placement and was sampled at a rate of 500 Hz. The electrodes were mounted in an elastic cap (Easy-Cap, Falk Minow, Herrching-Breitbrunn, Germany) and a SynAmps1 amplifier (Compumedics NeuroScan, Abbotsford, VIC, Australia) was used. The horizontal eye movements were recorded from electrodes at the outer canthus of each eye, and the vertical eye movements from electrodes placed above and below the right eye. Two additional electrodes were affixed at mastoid locations, and the ground electrode was placed on a cephalic site. The EEG was referenced online to the left mastoid. During EEG recording electrical impedances were kept below 5 kΩ.

#### Data Analysis

##### Digit span task

The percentage of accurate responses for the digit span task was calculated for each participant. The total numbers of digit sequences repeated correctly were collapsed across forward span and backward span. The participants were divided into *above average* and *below average* groups according to their accuracy percentages.

##### ERP experiment

The EEG data were processed offline using NeuroScan 4.3 EDIT software (Compumedics NeuroScan, Abbotsford, VIC, Australia). Data were band-pass filtered from 0.1 to 30 Hz (24 dB/oct; zero phase-shift). Eye blink artifacts were corrected using the ocular artifact reduction algorithm implemented in the Edit 4.3 software. The raw EEG data were then segmented into epochs of 1000 ms, with a 100 ms pre-stimulus baseline and 900 ms after the onset of the stimulus. The epoch data were arithmetically re-referenced to the average of both mastoids. Trials exceeding ±80 μV in any channel on the entire epoch were rejected. Finally, the ERPs were averaged separately for each stimulus type, electrode and participant. On average, 96 trials for each stimulus type were included in data analysis. The grand-averaged difference waves were generated for each stress pattern by subtracting the average responses to the clean standard stimuli from average responses to the corresponding deviant stimuli.

Based on visual inspection of the raw ERPs, mean amplitudes within six consecutive time windows of 100 ms were analyzed from 300 to 900 ms after stimulus onset. The mean amplitudes were computed for four regions: left-frontal (LF) included the electrodes F7, F3, FT7, and FC3; right-frontal (RF) included the electrodes F4, F8, FC4, and FT8; left-posterior (LP) included the electrodes TP7, CP3, P7, and P3; and right-posterior (RP) included the electrodes CP4, TP8, P4, and P8.

All the *p*-values and the *F*-values were adjusted using the Greenhouse-Geisser correction and the *post hoc* paired *t-*tests were adjusted using the Bonferroni correction for multiple comparisons.

### Results

#### Digit Span Task

Accuracy in the digit span task ranged from 29% to 71%, with an average of 52% (*SD* = 10.97%). The group classification was based on a median split for the accuracy results. Table 1 shows the number of participants in each group and their mean accuracy percentages. Independent samples *t*-test revealed that the two groups significantly differed from each other in the accuracy percentages [*t*(18) = 5.23, *p* < 0.001].

**Table 1:** Number of participants and mean accuracy percentages for the above average and below average groups in the digit span task.

Group	Number of participants	Mean accuracy percentage
Above average	10	60.2% (6.58%)
Below average	10	43.6% (7.59%)

*Standard deviations are in parentheses.*

#### ERP Data

**FIGURE 2:**
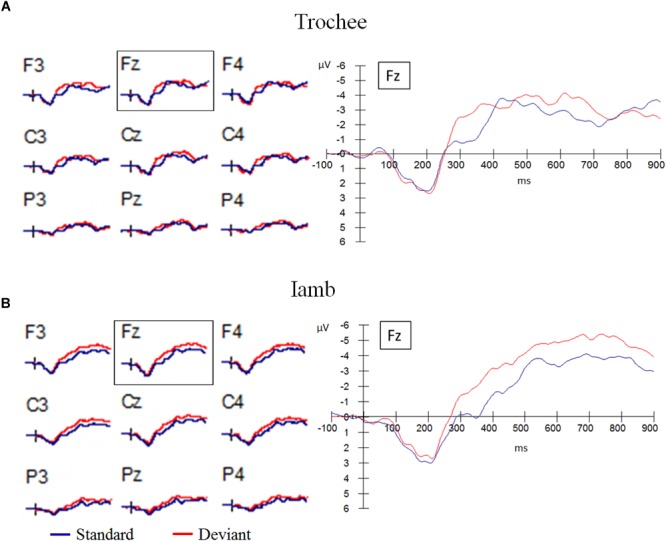
Grand averages of the frontal electrodes (F3, Fz, and F4), the central electrodes (C3, Cz, and C4) and the parietal electrodes (P3, Pz, and P4) for all participants. **(A)** Trochaic stress pattern. **(B)** Iambic stress pattern.

Grand averages of the frontal electrodes (F3, Fz, and F4), the central electrodes (C3, Cz, and C4) and the parietal electrodes (P3, Pz, and P4) are presented in Figure 2A for the trochaic stress pattern and in Figure 2B for the iambic stress pattern. A MMN component was elicited for the deviant versus standard stimuli, with a prominent frontal distribution between 300 and 400 ms for the trochaic stimuli, and between 300 and 500 ms for the iambic stimuli. A late negativity component was also observed at the frontal and central electrodes between 500 and 700 ms for the trochaic stimulus and between 500 and 900 ms for the iambic stimulus.

##### Trochee

The mean amplitudes for each stress pattern and latency window were submitted to 2 × 2 × 2 × 2 repeated measures ANOVAs with discrimination (deviant vs. standard), hemisphere (left vs. right), and anteriority (anterior vs. posterior) as within-subject factors and group (as defined by the auditory WM task: above average vs. below average) as between subject factor. The main effect of discrimination was significant in the time windows of 300–400 ms [*F*(1,18) = 13.67, *p* = 0.002, η^2^= 0.43] and 600–700 ms [*F*(1,18) = 5.08, *p* = 0.037, η^2^= 0.22]. A significant main effect of hemisphere was observed from 500 to 900 ms after the stimulus onset, with the amplitude being more negative in the right hemisphere than in the left hemisphere (500–600 ms: [*F*(1,18) = 4.51, *p* = 0.048, η^2^= 0.20]; 600–700 ms: [*F*(1,18) = 5.03, *p* = 0.038, η^2^= 0.22]; 700–800 ms: [*F*(1,18) = 9.47, *p* = 0.006, η^2^= 0.35]; 800–900 ms: [*F*(1,18) = 8.13, *p* = 0.011, η^2^= 0.31]). The main effect of anteriority was significant in all the six time windows, with frontal electrodes eliciting more negative amplitude (300–400 ms: [*F*(1,18) = 10.85, *p* = 0.004, η^2^= 0.38]; 400–500 ms: [*F*(1,18) = 22.24, *p* < 0.001, η^2^= 0.55]; 500–600 ms: [*F*(1,18) = 7.21, *p* = 0.015, η^2^= 0.29]; 600–700 ms: [*F*(1,18) = 9.13, *p* = 0.007, η^2^= 0.34]; 700–800 ms: [*F*(1,18) = 19.84, *p* < 0.001, η^2^= 0.52]; 800–900 ms: [*F*(1,18) = 38.68, *p* < 0.001, η^2^= 0.68]). In the time window of 300–400, there was a significant interaction of discrimination × hemisphere × anteriority [*F*(1,18) = 5.82, *p* = 0.027, η^2^= 0.24]. Paired samples *t*-tests showed that the discrimination effect was significant in all four regions: LF [*t*(19) = 4.47, *p* < 0.001], RF [*t*(19) = 2.56, *p* = 0.019], LP [*t*(19) = 2.33, *p* = 0.031], and RP [*t*(19) = 2.88, *p* = 0.01]. The interaction of discrimination × hemisphere × anteriority was also significant in the time windows of 400–500 ms [*F*(1,18) = 10.33, *p* = 0.005, η^2^= 0.37] and 500–600 ms [*F*(1,18) = 4.68, *p* = 0.044, η^2^= 0.21]. Further paired samples *t*-tests revealed that the discrimination effect was not significant in any of the four regions in these two time windows (*p*s > 0.05). In the time window of 600–700 ms, significant interactions of discrimination × anteriority [*F*(1,18) = 7.07, *p* = 0.016, η^2^= 0.28] and discrimination × hemisphere × anteriority [*F*(1,18) = 5.48, *p* = 0.031, η^2^= 0.23] were observed. Paired samples *t*-tests revealed that the discrimination effect was only significant in the LF region [*t*(19) = 3.49, *p* = 0.002], and marginally significant in the RF region [*t*(19) = 2.07, *p* = 0.053]. No significant effect or interaction of group was found.

##### Iamb

A significant main effect of discrimination was observed from 300 to 800 ms after the stimulus onset (300–400 ms: [*F*(1,18) = 26.07, *p* < 0.001, η^2^= 0.59]; 400–500 ms: [*F*(1,18) = 14.82, *p* = 0.001, η^2^= 0.45]; 500–600 ms: [*F*(1,18) = 9.80, *p* = 0.006, η^2^= 0.35]; 600–700 ms: [*F*(1,18) = 9.84, *p* = 0.006, η^2^= 0.35]; 700–800 ms: [*F*(1,18) = 5.60, *p* = 0.029, η^2^= 0.24]). Moreover, the main effect of anteriority was significant in all the six time windows, with the negativity being more prominent in the frontal area (300–400 ms: [*F*(1,18) = 35.47, *p* < 0.001, η^2^= 0.66]; 400–500 ms: [*F*(1,18) = 46.46, *p* < 0.001, η^2^= 0.72]; 500–600 ms: [*F*(1,18) = 56.02, *p* < 0.001, η^2^= 0.76]; 600–700 ms: [*F*(1,18) = 112.80, *p* < 0.001, η^2^= 0.86]; 700–800 ms: [*F*(1,18) = 200.84, *p* < 0.001, η^2^= 0.92]; 800–900 ms: [*F*(1,18) = 98.38, *p* < 0.001, η^2^= 0.85]). In the time window of 300–400 ms, there was a significant main effect of group [*F*(1,18) = 6.66, *p* = 0.019, η^2^= 0.27] and a significant interaction of discrimination × group [*F*(1,18) = 4.93, *p* = 0.039, η^2^= 0.22]. Further analysis showed that the discrimination effect was more significant in the *above average* group [*F*(1,9) = 22.27, *p* = 0.001, η^2^= 0.71] than in the *below average* group [*F*(1,9) = 5.24, *p* = 0.048, η^2^= 0.37]. In the time window of 400–500 ms, a main effect of hemisphere was found, with the negativity being more prominent in the right hemisphere than in the left hemisphere [*F*(1,18) = 4.61, *p* = 0.046, η^2^= 0.20]. There was a significant interaction of discrimination × anteriority for the time window of 700–800 ms [*F*(1,18) = 9.14, *p* = 0.007, η^2^= 0.34]. Paired samples *t*-tests only yielded significant discrimination effects in the frontal region [*t* (19) = 3.00, *p* = 0.007], but not in the parietal region [*t*(19) = 1.64, *p* = 0.12].

##### Difference wave

**FIGURE 3:**
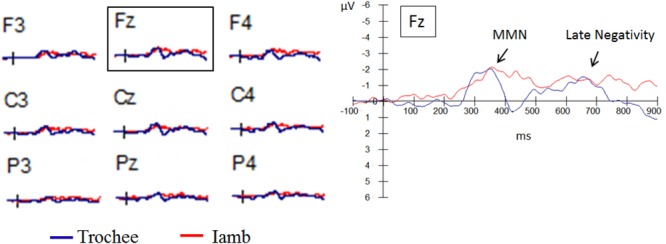
Grand-average difference waves (deviant minus standard) of the frontal electrodes (F3, Fz, and F4), the central electrodes (C3, Cz, and C4) and the parietal electrodes (P3, Pz, and P4) for the trochaic and iambic stress patterns.

**FIGURE 4:**
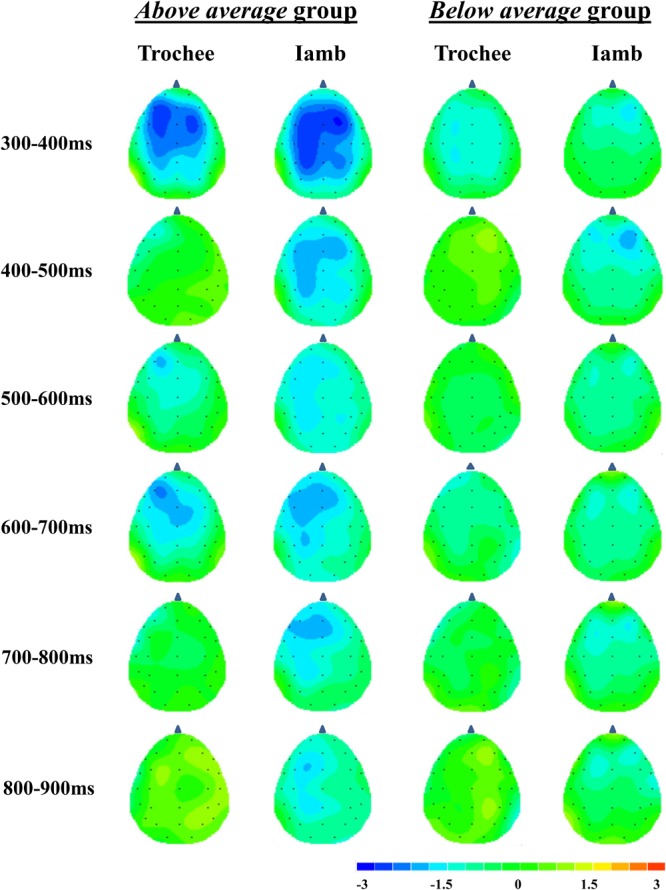
Topographical isovoltage maps obtained on difference waves (mean amplitude in the time windows 300–400, 400–500, 500–600, 600–700, 700–800, and 800–900 ms) of the trochaic and iambic stress patterns for the *above average* and *below average* groups.

Figure 3 displays the grand-average difference waves (deviant minus standard) for the trochaic and iambic stresses. Figure 4 presents the topographical isovoltage maps in the six time windows for the two stress patterns, by the two groups established in the auditory WM task.

In order to directly compare the differences between the trochaic and iambic conditions, we further performed six 2 × 2 × 2 × 2 repeated measures ANOVAs on the difference waves for the six time windows, with stress (trochee vs. iamb), hemisphere (left vs. right), and anteriority (anterior vs. posterior) as within-subject factors and group (above average vs. below average) as between subject factor. The results yielded significant main effect of stress in the time windows of 400–500 ms [*F*(1,18) = 7.61, *p* = 0.013, η^2^= 0.30] and 800–900 ms [*F*(1,18) = 5.19, *p* = 0.035, η^2^= 0.22], with the difference wave being more negative in the iambic condition than in the trochaic condition. A significant main effect of anteriority was observed in the time windows of 300–400 ms [*F*(1,18) = 10.52, *p* = 0.005, η^2^= 0.37], 600–700 ms [*F*(1,18) = 12.12, *p* = 0.003, η^2^= 0.40], and 700–800 ms [*F*(1,18) = 8.23, *p* = 0.010, η^2^= 0.31], with the anterior region showing larger negativities than the posterior region. Moreover, in the time window of 300–400 ms, a significant main effect of group was found [*F*(1,18) = 5.18, *p* = 0.035, η^2^= 0.22]. The difference wave was more negative in the *above average* group than in the *below average* group. However, the interaction of stress × group was not significant [*F*(1,18) = 0.36, *p* = 0.56], suggesting that regardless of stress pattern the *above average* group was better at stress processing than the *below average* group. Table 2 summarizes the main effects and interactions in the six time windows of 100 ms for the trochaic and iambic condition and the difference wave.

**Table 2:** Main effects and interactions for the behavioral and ERP data.

	300–400	400–500	500–600	600–700	700–800	800–900
**A. Trochee**						
Discrimination (Dis)	^∗∗^			^∗^		
Hemisphere (Hem)			^∗^	^∗^	^∗∗^	^∗^
Anteriority (Ant)	^∗∗^	^∗∗∗^	^∗^	^∗∗^	^∗∗∗^	^∗∗∗^
Dis × Hem		^∗^				
Dis × Ant				^∗^		
Dis × Hem × Ant	^∗^	^∗∗^	^∗^	^∗^		
**B. Iamb**						
Dis	^∗∗∗^	^∗∗^	^∗∗^	^∗∗^	^∗^	
Hem		^∗^				
Ant	^∗∗∗^	^∗∗∗^	^∗∗∗^	^∗∗∗^	^∗∗∗^	^∗∗∗^
Group	^∗^					
Dis × Group	^∗^					
Dis × Ant					^∗∗^	
Hem × Ant						^∗^
**C. Difference wave**		
Stress (Trochee		^∗^				^∗^
vs. Iamb)
Group	^∗^					
Ant	^∗∗^			^∗∗^	^∗^	
Hem × Ant		^∗^			^∗^	

*See text for degrees of freedom, *F*, and *p-*values. ^∗^*p* < 0.05, ^∗∗^*p* < 0.01, ^∗∗∗^*p* < 0.001.*

## Experiment 2

In Experiment 2, we conducted an ABX discrimination task to investigate the EP speakers’ attentive stress processing in the absence of vowel quality cues. The experimental procedure was almost the same as the experiment 1 in Correia et al. (2015), except that they used both disyllabic and trisyllabic non-sense words as stimuli while we only included disyllabic non-sense words.

### Methods

#### Participants

Participants were the same as in Experiment 1.

#### Materials

In the behavioral task, stimuli consisted of eight disyllabic non-sense words ([diku], [diru], [midu], [misu], [kiru], [kisu], [siru], and [sisu]) associated with either a trochaic or an iambic stress^3^. The stimuli were produced by one male and two female native EP speakers in a sound-attenuated booth. All of these non-sense words are legal syllable structures in EP, thus participants could focus on the stress patterns and not be distracted by unfamiliar syllables. All stimuli included the high vowels [i] and [u], because high vowels do not show vowel reduction in unstressed positions in EP. In addition, EP words end in [u], both stressed and unstressed, are more common than their counterparts end in [i].

#### Procedure

The behavioral discrimination task used a forced-choice ABX paradigm, in which the participants heard three stimuli in a series and had to decide whether the third stimulus X had the same stress pattern as the first stimulus A or the second stimulus B by pressing the ‘z’ (stimulus A) and ‘m’ (stimulus B) keys on a keyboard. The stimuli A and B might be produced by the same female speaker or by two different female speakers, and always had contrasting stress patterns. The stimulus X was always produced by a male speaker. In each trial the three stimuli were separated by 500 ms inter-stimulus-interval. A total of 144 experimental trials were presented, with all 16 possible combinations.^4^ The experimental trials were randomized for each participant, and were preceded by four practice trials which were excluded from data analysis. The participants were encouraged to respond as quickly and accurately as possible. Their accuracies and reaction times were logged by the E-prime 2.0 software (Schneider et al., 2012). No feedback was given through the task. A break of at least 30 s was given halfway through the task, in order to prevent fatigue.

#### Data Analysis

Data obtained from the ABX task were converted to d’ scores, which were calculated as the difference between the z-transforms of hit rates and false alarm rates (Macmillan and Creelman, 2005). The hit rates were calculated as correct matches of X to the first stimulus (i.e., AB-A and BA-B), and false alarms as incorrect matches of X to the second stimulus (i.e., AB-B, BA-A). Furthermore, the error rate for each combination was calculated for each participant.

Participants’ reaction times (RTs) were computed for the correct trials. RTs that were greater than the mean RT of all the correct responses plus 2.5 standard deviations for an individual participant were replaced by this value. After the outlier replacement, the mean RT for each combination was calculated for each participant.

### Results

The mean d’ scores in the ABX task for the *above average* and *below average* groups were 1.73 (*SD* = 0.52) and 1.69 (*SD* = 0.82), respectively. Independent samples *t*-test showed that the two groups did not differ from each other on d’ scores [*t*(18) = 0.14, *p* = 0.89].

**FIGURE 5:**
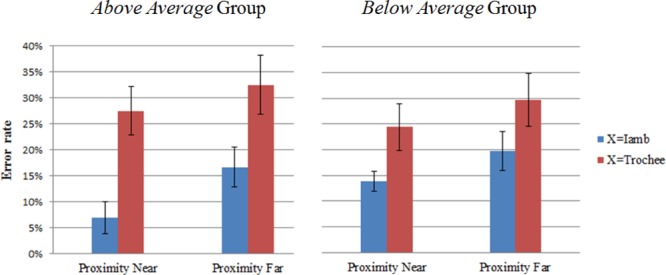
Mean error rates with standard errors of the ABB, BAA, ABA, and BAB combinations in the ABX task for the two groups.

In order to examine which combination resulted in more errors (i.e., ABB, BAA, ABA, and BAB), we further calculated mean error rates for each combination. Figure 5 presents the mean error rates of each combination in the ABX task. The mean error rate across the four combinations was 21.42% (*SD* = 7.66%), which replicates the result of the ABX experiment reported in Correia et al. (2015). A repeated measures ANOVA was performed on the error rates, with proximity (near: ABB and BAA vs. far: ABA and BAB) and stress (X = trochee vs. X = iamb) as within-subject factors, and group (above average vs. below average) as between subject factor. The results revealed a marginally significant main effect of proximity [*F*(1,18) = 4.39, *p* = 0.050, η^2^= 0.20], with ‘near’ combinations showing less errors than the ‘far’ combinations. Moreover, there was a significant main effect of stress [*F*(1,18) = 19.36, *p* < 0.001, η^2^= 0.52]. The participants’ responses were more accurate if the X stimulus had an iamb stress than if the X stimulus had a trochee stress. No other significant main effect or interaction was found.

**FIGURE 6:**
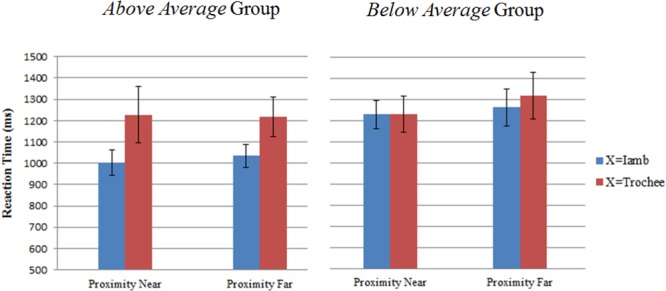
Mean reaction times with standard errors of the ABB, BAA, ABA, and BAB combinations in the ABX task for the two groups.

The mean reaction times of all the correct responses for the *above average* and *below average* groups were 1,097 ms (*SD* = 222 ms) and 1,255 ms (*SD* = 263 ms), respectively. Figure 6 shows the mean reaction times of each combination in the ABX task. The reaction times were submitted to a 2 × 2 × 2 repeated measures ANOVA, with proximity and stress as within-subject factors and group as between-subject factor. A significant main effect of stress was observed [*F*(1,18) = 7.50, *p* = 0.01, η^2^= 0.29]. Participants responded more quickly if the X stimulus had an iambic stress than if the X stimulus had a trochaic stress. The interaction of stress × group was marginally significant [*F*(1,18) = 4.20, *p* = 0.055, η^2^= 0.19]. Independent samples *t*-test showed that the participants in the *above average* group responded more quickly than the participants in the *below average* group if the X stimulus had an iambic stress [*t*(18) = -2.56, *p* = 0.02]. However, if the X stimulus had a trochaic stress, the two groups did not differ from each other in response time [*t*(18) = -0.37, *p* = 0.72]. No other significant main effect or interaction was observed.

### Cross-Experiment Analysis

The Pearson correlation coefficient between the electrophysiological measures from Experiment 1 and the behavioral measures from Experiment 2 was calculated for the trochaic and iambic conditions separately. More specifically, the correlations were calculated on the basis of each participant’s accuracy in the digit span task, MMN amplitude, late negativity amplitude, error rate, and reaction time. Since the negativity effects were more prominent in the frontal area in the ERP task, the Fz electrode was chosen for the calculation.

**FIGURE 7:**
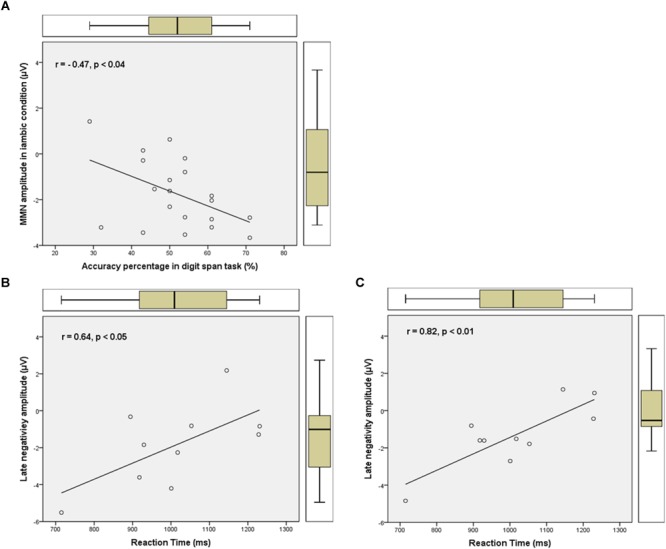
Pearson correlation coefficient between electrophysiological measures from Experiment 1 and behavioral measures from Experiment 2. **(A)** Negative correlation between the MMN amplitude in the 300–400 ms in the iambic condition and the participants’ accuracy percentage in the digit span task. **(B,C)** Positive correlation between the late negativity amplitudes in the 700–800 ms and 800–900 ms time windows in the iambic condition and the reaction time when the X stimulus had an iambic stress in the ABX task for the *above average* group.

Correlation analyses showed that in the iambic condition the MMN amplitude in the 300–400 ms time window was significantly correlated with the participants’ accuracy percentage in the digit span task. The higher the participants’ WM capacity, the larger MMN amplitude (i.e., more negative) they displayed in the iambic condition (*r* = -0.47, *p* = 0.035). Moreover, for the *above average* group the late negativity mean amplitudes of the 700–800 ms (*r* = 0.64, *p* = 0.047) and 800–900 ms (*r* = 0.82, *p* = 0.004) time windows in the iambic condition were significantly correlated with the mean reaction time when the X stimulus had an iambic stress in the ABX task: the larger the late negativity amplitude, the shorter the reaction time. No other significant correlations were found for the *above average* group or for the *below average* group. Scatter plots showing the positive and negative correlations were presented in Figure 7.

## Discussion

In the present study, we recorded native EP speakers’ electrophysiological and behavioral data to examine (1) whether they could attentively and pre-attentively discriminate CVCV non-sense words with trochaic and iambic stress patterns in the absence of vowel quality cues; (2) whether they would show asymmetrical performance for the trochaic and iambic stress patterns; and (3) how WM abilities would influence their stress processing at the behavioral and electrophysiological levels.

In the ERP task, both the trochaic and iambic conditions yielded MMN and late negativity effects. The components in the iambic condition are more negative and span over a larger temporal window than in the trochaic condition. Although in the iambic condition the participants with high WM capacity showed a more prominent discrimination effect than those with low WM capacity, the analyses on difference waves revealed that overall the participants with high WM capacity exhibited a more prominent discrimination effect regardless of stress pattern. In the behavioral task, the participants with high and low WM capacity did not differ from each other on discrimination accuracy. Overall, the EP participants made 21% errors in the ABX task. This high error rate speaks in favor of stress “deafness” by EP speakers in the absence of vowel quality cues. In addition, the behavioral data revealed an iambic advantage: EP speakers responded more accurately and more quickly if the X stimulus had an iambic stress than if the X stimulus had a trochaic stress. In the following, we will discuss our findings according to the three main research questions addressed in the present study.

### EP Speakers’ Perception of Stress in the Absence of Vowel Quality Cues

European Portuguese is a language with variable stress. Previous research has identified vowel quality as the primary cue for the perception of stress in EP. Correia et al. (2015) reported that if the vowel reduction cue was removed EP speakers demonstrated a stress “deafness” effect similar to that found in speakers of languages with fixed stress. Correia et al. (2015) followed the same procedures as described in Dupoux et al. (1997) and performed an ABX task on EP participants. In Dupoux et al.’s (1997) experiment, French participants (fixed stress pattern) made 20% errors in the stress contrast, whereas Spanish participants (variable stress pattern) made only 4% errors. Because the performance between the two populations was significantly different, Dupoux et al. (1997) claimed that French listeners are “deaf” to stress contrasts even though they performed much better than chance (50%). In Correia et al. (2015), EP participants made 21% errors, which is comparable with the errors made by the French participants in Dupoux et al. (1997). Our behavioral data again replicates Correia et al.’s (2015) results, showing that EP participants made 21% errors in the ABX task without vowel reduction cue. These results suggest that stress “deafness” may not be specific to languages with fixed stress, but rather a perceptual inability that emerges when the critical acoustic cue was absent. The EP participants could perceive the difference between the two types of stimuli using some acoustic-based strategies, but these strategies are not enough on a meta-linguistic level (Domahs et al., 2012; Rahmani et al., 2015). In other words, EP listeners failed in the behavioral discrimination task because the perceptual cues available (i.e., the prosodic cues) are not sufficient to match their phonological representations of stress at an attentive level.

In the ERP task, both the trochaic and iambic conditions yielded MMN and late negativity effects, indicating that native EP speakers are able to discriminate the two stress patterns without vowel reduction at the pre-attentive stage. The behavioral and ERP results were consistent with previous studies on Turkish which also yielded divergent outcomes between the behavioral and ERP methods (Altmann, 2006; Domahs et al., 2013). One may argue that the discrepancy between the behavioral and electrophysiological results in the present study was due to the fact that in the behavioral task we used a variety of disyllabic combinations while in the ERP task we only included exemplars of one disyllable. The lack of syllabic variation in the ERP task resulted in a fine-grained situation, which could make it easy for the EP speakers to use constant acoustic properties to discriminate the two stress patterns. However, we only partly agree with this explanation because in the present study we actually included two tokens of each stress pattern and thus provided some evidence that EP speakers are able to pre-attentively discriminate the two stress types in the absence of vowel quality cues on the basis of some higher level category representations. In future studies, it would be interesting to use varied disyllabic combinations in the ERP task to explore whether EP speakers can encode implicit and abstract auditory rules of stress patterns at the pre-attentive stage.

### Asymmetrical Stress Perception by EP Speakers

According to previous studies (as reviewed in the Introduction section, e.g., Jusczyk et al., 1993; Crosswhite et al., 2003; Weber et al., 2004; Friederici et al., 2007; Höhle et al., 2009; Domahs et al., 2012, 2013; Molczanow et al., 2013), we made two predictions regarding the asymmetrical processing of stress by EP speakers: if EP speakers behave similarly to the native speakers of languages with fixed stress, they may not show advantage for either the trochaic or iambic stress pattern behaviorally, but may display an increased sensitivity to the less frequent stress pattern at the electrophysiological level; However, if EP speakers behave similarly to the speakers of languages with variable stress, they may exhibit an advantage for the more frequent stress pattern in the behavioral task, and demonstrate either MMNs for both stress patterns, or a preference for one of the stress patterns in the ERP task.

In the present study, both the behavioral and ERP data revealed a processing advantage for the iambic stress pattern. EP speakers responded more accurately and more quickly if the X stimulus had an iambic stress than if the X stimulus had a trochaic stress in the ABX task. In the ERP task, both the MMN and the late negativity components were more negative and span over a larger temporal window in the iambic condition than in the trochaic condition. Many training studies have shown that behavioral improvements in speech perception are usually accompanied by an increased MMN (e.g., Tremblay et al., 1997; Menning et al., 2002; Näätänen et al., 2004; Kaan et al., 2007). Kraus et al. (1995) found that after a behavioral discrimination training participants’ MMN response increased not only in magnitude but also in duration. Therefore, we argue that the more prominent and prolonged negativity effects in the current study reflect an ease of processing of the iambic stress pattern. In addition, our findings are compatible with a recent study on stress processing by native EP infants. Using an Anticipatory Eye Movement (AEM) paradigm, Butler et al. (2015, unpublished) found that 5- to 6-month-old EP-learning infants looked longer at the side associated with the iambic stress pattern than the side associated with the trochaic stress pattern. These behavioral and electrophysiological results on EP adults and infants seem to indicate that the iambic stress pattern is perceptually more salient and thus more easily discernible for EP speakers (Friederici et al., 2002; Weber et al., 2004; Klein et al., 2011). There are two possible explanations that may account for this processing advantage of the iambic stress pattern. First, the processing of word stress in EP has been claimed to occur in a leftward fashion (Mateus and Andrade, 2000; Vigário, 2003). From a cognitive perspective, processing costs should increase with increasing distance of stress position from the starting point of computation (e.g., Domahs F. et al., 2014). Since stress computation works from right to left in EP, penultimate stress should be computationally more demanding than ultimate stress. This may explain why we found an advantage for iambic stress in both the behavioral and ERP tasks in the present study. The second account is based on the statistical distribution of stress patterns in EP. As discussed in the “Introduction” section, if we consider token frequency and add monosyllabic words to the computation of the final stress pattern, final stress becomes the most frequent stress pattern in EP (Vigário et al., 2010). Therefore, it is detected more easily by EP infants and adult speakers. Our results, in return, provide evidence for an iambic prosodic default in EP.

In sum, in the absence of vowel quality cues, EP speakers still behave similarly to speakers of languages with variable stress, demonstrating a processing advantage for the predominant stress pattern at both the behavioral and electrophysiological levels. Moreover, this perceptual bias for stress seems to emerge very early in development and may become part of the architecture of stress phonological systems (Polizer-Ahles et al., 2016).

### WM Abilities and Stress Processing

In the behavioral task, EP speakers with high WM capacity responded more quickly than those with low WM capacity if the X stimulus had an iambic stress, but not when the X stimulus had a trochaic stress. In the ERP task, the participants with high WM capacity exhibited a more prominent MMN regardless of stress pattern. Moreover, our correlation analyses for the *above average* WM group revealed that in the iambic condition the prolonged late negativity amplitude was positively correlated with the reaction time when the X stimulus had an iambic stress in the ABX task: the larger the late negativity amplitude, the shorter the reaction time. Using a non-word reading task, Domahs F. et al. (2014) failed to find a significant correlation between WM capacity and stress assignment to the penultimate syllable, but they showed that German participants with good WM capacity demonstrated a processing advantage for the cognitively more demanding stress pattern (i.e., antepenultimate stress). In the present study, EP speakers with high WM capacity revealed an overall advantage at the electrophysiological level, but only a processing advantage for the computationally less demanding stress pattern at the behavioral level. As suggested by Tremblay et al. (1998), neural processing may be measured before functional behavior. In other words, the neurophysiological measures might be more sensitive than the behavioral measures to show the processing advantages by the EP speakers with high WM capacity. Therefore, we found a more salient MMN not only for the “default” stress but also for the computationally more demanding stress pattern in the participants with good WM. However, the behavioral measures only revealed a processing advantage for the computationally less demanding stress pattern. In addition, the behavioral ABX task involved a change in talker (i.e., the stimuli A and B were produced either by the same female speaker or by two different female speakers; and the stimulus X was always produced by a male speaker), which might require the participants to use a more abstract level of representation in order to retain the relevant acoustic information in a short term memory store. This might have posed an additional challenge to the processing of computationally more demanding stress pattern even for the EP speakers with high WM capacity.

## Conclusion

Using psychophysiological and behavioral measures, the present study demonstrated that native EP speakers can pre-attentively discriminate CVCV pseudo-words with trochaic and iambic stress patterns in the absence of vowel quality cues. These results argue against stress “deafness” in EP, and suggest the need of a multi-methodological approach to stress processing. Moreover, our ERP and behavioral data revealed a processing advantage for the iambic stress pattern, arguing in favor of an iambic prosodic default in EP.

## Ethics Statement

The study was carried out in accordance with the recommendations of ‘European Union Agency for Fundamental Rights’ with written informed consent from all subjects, following Portuguese regulations. As part of the EBELa project (EXCL/MHC-LIN/0688/2012), the study was approved by the Ethics Committee for Health of Centro Hospitalar Lisboa Norte, and by the Ethics Committee of the Regional Health Administration Lisboa e Vale do Tejo, Portugal.

## Author Contributions

SF, MV, and SL conceived and designed the experiments. SL performed the experiments and analyzed the data. SC and RJ contributed to data collection. SL and SF wrote the paper.

## Conflict of Interest Statement

The authors declare that the research was conducted in the absence of any commercial or financial relationships that could be construed as a potential conflict of interest.

## Footnotes

1 Indeed, vowel quality is highly correlated with stress position in EP: stressed syllables always exhibit unreduced vowels, whereas unstressed syllables may show reduced vowels (the most common pattern due to phonological vowel reduction) or unreduced ones. A computation of the distribution of reduced and unreduced vowels in unstressed positions, using the 5,294 most frequent word types from the FrePOP Lexicon (Vigário et al., 2015) which represent a corpus of over 2.6 million tokens, shows that 91.5% of all vowels in unstressed position are reduced vowels.

2 According to the database of FrePOP, monosyllabic words and trisyllabic and longer words appear in similar proportions (28.6% and 27%, respectively). There are two main reasons why we should consider monosyllabic words as words with final stress: (1) word stress is computed from the right edge of the word in EP (Mateus and Andrade, 2000; Vigário, 2003); (2) monosyllabic words share many properties with stress-final syllables (Vigário et al., 2006).

3 The stimuli in the present behavioral task are a selection of the stimuli in Experiment 1 of Correia et al. (2015).

4 The 16 possible combinations are: A_1_B_1_-A_3_, A_1_B_2_-A_3_, A_2_B_1_-A_3_, A_2_B_2_-A_3_, A_1_B_1_-B_3_, A_1_B_2_-B_3_, A_2_B_1_-B_3_, A_2_B_2_-B_3_, B_1_A_1_-A_3_, B_1_A_2_-A_3_, B_2_A_1_-A_3_, B_2_A_2_-A_3_, B_1_A_1_-B_3_, B_1_A_2_-B_3_, B_2_A_1_-B_3_, and B_2_A_2_-B_3_. The letters represent the stress patterns, and the numbers represent the speakers.
